# Clinical presentation, diagnostic findings and management of cerebral ischemic events in patients on treatment with non-vitamin K antagonist oral anticoagulants – A systematic review

**DOI:** 10.1371/journal.pone.0213379

**Published:** 2019-03-29

**Authors:** Thomas Raphael Meinel, Sebastién Frey, Marcel Arnold, Sarah Kendroud, Urs Fischer, Johannes Kaesmacher, Mirjam Rachel Heldner, Simon Jung

**Affiliations:** 1 Department of Neurology, Inselspital, Bern University Hospital, University of Bern, Bern, Switzerland; 2 Department of Neurology, Touro University California, Vallejo, California, United States of America; 3 Department of Diagnostic and Interventional Neuroradiology, Inselspital, Bern University Hospital, University of Bern, Bern, Switzerland; Texas Biomedical Research Institute, UNITED STATES

## Abstract

**Background:**

Non-vitamin K antagonist oral anticoagulants (NOAC) are equally or potentially superior in terms of effectiveness in the prevention of ischemic stroke and carry a lower associated risk of intracranial hemorrhage compared to Vitamin K antagonists. Nevertheless, ischemic strokes also occur in patients who are being treated with NOAC. In those particular patients, knowledge about the underlying stroke etiology, clinical presentation, acute management, and complication rates is scarce.

**Objective:**

Systematic literature review to provide a comprehensive clinical overview in terms of presentation, laboratory, imaging parameters and outcomes of patients suffering from acute cerebral ischemic events (i.e. TIA and acute ischemic stroke) while on treatment with a NOAC. Only if available, comparison to VKA is presented which was not the primary focus of this analysis.

**Data sources:**

PubMed/MEDLINE, Scopus and EMBASE from January 1, 2006, to November 20, 2018.

**Study eligibility criteria:**

52 studies providing detailed information on a total of 12247 patients were included. We excluded case reports and case series with less than five patients.

**Study appraisal and synthesis method:**

We systematically assessed study quality using a bias tool and pooled consistent data.

**Results:**

Existing data indicates milder stroke severity and smaller infarct size of acute ischemic stroke on treatment with NOAC compared to stroke occurrence on Vitamin K antagonists (VKA). Established risk factors for ischemic events also play a role in stroke while on NOACs, albeit the underlying etiology remains poorly understood. Intravenous thrombolysis and endovascular therapy seem to be safe and effective, but patient selection for recanalization therapies is challenging.

**Limitations:**

Limited quality of published data, duplicate cases, statistical issues of data pooling, possible incomplete retrieval of identified research and reporting bias might have limited our findings.

**Conclusions:**

Acute ischemic events despite treatment with NOAC therapy are insufficiently investigated.

**Systematic review registration number:**

PROSPERO: CRD42018074853.

## Introduction

### Rationale

The introduction of rivaroxaban in 2008 and the subsequent addition of three more non-vitamin K antagonist oral anticoagulants (NOAC) had an enormous impact on the primary and secondary prevention of acute ischemic stroke (AIS) in the setting of nonvalvular atrial fibrillation (AF). Most guidelines now recommend NOAC over vitamin K antagonists (VKA) with a class I recommendation level [1–4]. The net clinical benefit arises mainly from a reduced risk of intracerebral hemorrhage (ICH) with NOAC treatment, whereas the prevention of AIS or TIA, which are later in this review referred to as acute ischemic events (AIE), is equal or better compared to VKA treatment. Nevertheless, 1–2% of patients per year suffer from an AIE while on NOAC treatment [5]. The knowledge about stroke subtype, vessel occlusion location, pattern of cerebral infarction, complications and therapy strategies in those patients is scarce. Identifying factors that are associated with AIE that occur while on NOAC treatment may improve individualized treatment decisions. Furthermore, it is important to analyze the reasons for NOAC failure in real-life data, because randomized-controlled trials represent a highly selective patient population. For example an additional indication for anticoagulation, chronic non-steroidal anti-inflammatory drug therapy, severe chronic renal insufficiency, anemia, unreliability or short life expectancy (e.g. malignancy) were exclusion criteria in the pivotal phase III trials, but are likely to be present in NOAC real-life patients [6–9].

### Objectives

We aimed to provide a comprehensive clinical overview (clinical presentation, laboratory, imaging parameters and outcomes) of AIE in patients on NOAC treatment by performing a systematic review of the current literature. Only if available, comparison to VKA is presented which was not the primary focus of this analysis.

## Methods

### Protocol and registration

The study protocol has been published prior to performing the study (PROSPERO: CRD42018074853).

### Eligibility criteria

Eligible studies included all age groups and all ethnic groups with given demographic, clinical, laboratory or imaging information on NOAC patients in the setting of AIE. We included all evaluative methodologies. Since the first NOAC, rivaroxaban, entered the market in 2008, we only considered publications beyond January 1st, 2006 for analysis. We excluded studies with no specific characterization of patients who suffered an AIE while taking NOAC. We excluded case reports and case series with less than five patients.

### Information sources

Data for this review was identified via a search on MEDLINE, PubMed, Scopus and Embase and references from relevant articles using the search terms "DOAC" OR “NOAC” OR "anticoagulation", and "ischemic stroke" or “TIA”. Only articles published in English, French, Spanish and German between January 1^st^, 2006 and November 20^th^, 2018 were included.

### Search

This is the detailed search strategy used for PubMed: ("noacs"[All Fields] OR "noac"[All Fields] OR "non-vitamin k oral anticoagulants"[All Fields] OR "non-vka oral anticoagulant"[All Fields] OR "doacs"[All Fields] OR "doac"[All Fields] OR "direct oral anticoagulant"[All Fields] OR "direct oral anticoagulants"[All Fields] OR "factor xa inhibitor"[All Fields] OR "direct thrombin inhibitor"[All Fields] OR "dabigatran"[All Fields] OR "pradaxa"[All Fields] OR "rivaroxaban"[All Fields] OR "xarelto"[All Fields] OR "apixaban"[All Fields] OR "eliquis"[All Fields] OR "edoxaban"[All Fields] OR "lixiana"[All Fields] OR "savaysa"[All Fields]) AND ("ischemic stroke"[All Fields] OR "tia"[All Fields] OR "transient ischemic attack"[All Fields]) AND (("2006/01/01"[PDAT]: "2018/11/20"[PDAT]) AND "humans"[MeSH Terms] AND (German[lang] OR English[lang] OR French[lang] OR Spanish[lang])).

### Study selection

Citations were uploaded into the covidence online review tool. Their relevance was assessed against the predetermined inclusion and exclusion criteria by TRM and SF, who independently screened all titles and abstracts. Forward and backward reference searching complemented the database searches. Full-text manuscripts were obtained for all studies entering the review. Any uncertainties about including a specific manuscript in the review were resolved by consensus.

### Data collection process

Data was extracted onto an Excel spreadsheet by SF and reviewed by TRM.

### Data items

Data items included number of patients, age, sex, race, vascular risk factors, type of NOAC and dose, concomitant medication, clinical features of the AIE such as stroke severity, drug levels, renal function, coagulation parameters, imaging findings, stroke etiology, acute management and outcome parameters (functional outcome, mortality).

### Risk of bias in individual and across studies

We compared data items, outcomes, design strengths and weaknesses across the studies. For each study, the risk of bias was thoroughly assessed at the study level using a modified National Heart, Lung, and Blood Institute (NHLBI) bias tool, and this information was incorporated when interpretation of data was given in the synthesis.

### Summary measures

The principal summary measures were the clinical characteristics of patients using a NOAC in the setting of AIE.

### Synthesis of results

If available and consistent throughout the studies, the pooled mean weighted corresponding to the sample size is presented. Data items given by median and interquartile range were converted assuming gaussian distribution as described earlier [10]. Until stated otherwise, the comparison to VKA patients is provided within each individual study.

### Additional analyses

No additional analyses were performed.

### Study selection

**Fig 1:**
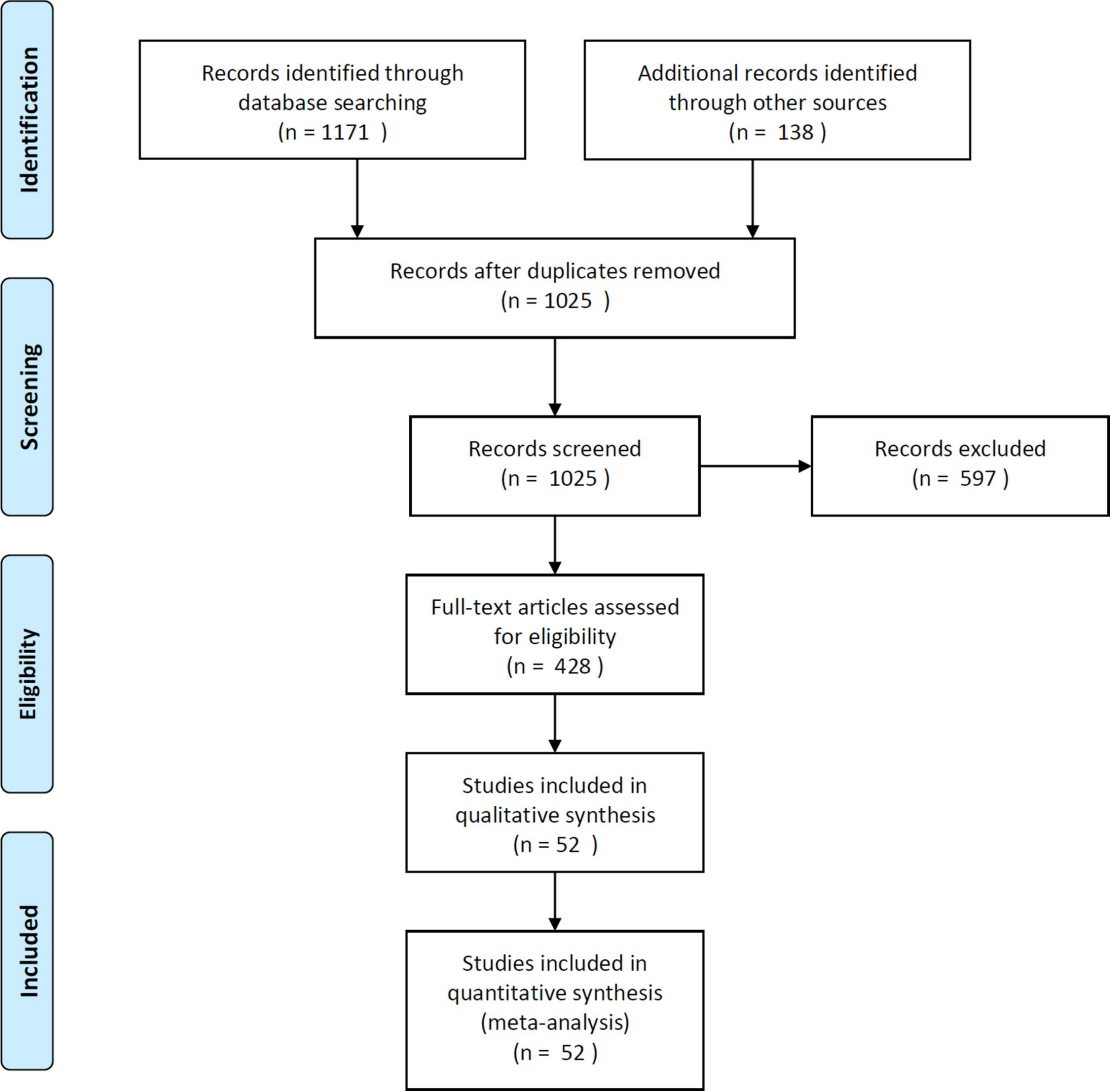
PRISMA flow diagram. Mainly studies were excluded because they only reported incidence rates of ischemic stroke or TIA patients without further clinical information on those patients.

The database searches and citation tracking yielded 1309 hits, of which 1025 records were screened as potentially relevant after removing duplicates (Fig 1). Reasons for excluding relevant publications were mainly due to the lack of specific data items on individual patients. In total, 52 publications including 12247 patients met the inclusion criteria and were included in the analysis (1 substudy of a randomized interventional trial, 1 prospective non-randomized interventional trial, 1 nested case-control study, 28 non-randomized observational studies; 18 case series, 2 diagnostic studies, 1 substudy of an observational register).

### Study characteristics

The extracted data of the included publications is shown in S1 Table.

### Risk of bias within and across studies

We used 14 quality items to assess bias using a modified NHLBI bias tool [11]. We defined study quality as low when the sum of the positive quality items was less than six and moderate when it was between six and nine. Overall, the risk of bias was considerable across the studies. 20 studies had moderate quality, and 32 studies had low quality. Retrospective design, small sample size, publication bias, selective reporting and the slanted choice of a particular NOAC therapy versus VKA therapy contributed to bias. The detailed bias assessment of each individual study using the modified NHLBI bias tool is available in the S2 Table.

## Results

### Risk factors and reasons for AIE while using a NOAC

In patients starting NOAC therapy, AIE were reported to occur more frequently during the first three months of treatment [12,13] with a declining AIE rate thereafter. Established risk factors such as previous AIE or an elevated CHA2DS2-VASc score were reported to predict AIE while using a NOAC [14–16]. Renal impairment, defined as estimated glomerular filtration rate of less than 60ml/min, was also reported to be a predictor of AIS while on NOAC therapy [17], which fits the borderline impaired pooled renal function parameters in our cohort. Kamal et al. reported an elevated mean body mass index (BMI) in NOAC failure [18]. This is in contrast to Arihiro et al. who found a reduced BMI patients with AIE in NOAC failure [19]. The mean weighted BMI in the pooled cohort was slightly elevated.

#### Severity and infarction size of AIE

Overall, AIS severity while on a NOAC measured by the National Institutes of Health Stroke Scale (NIHSS score) was mostly mild, and the outcome was favorable compared to AIS severity while on a VKA [13,15,20–27]. The mean weighted NIHSS score on admission in the pooled NOAC cohort was 4.6. In the biggest data set from the *Get with the Guidelines Register*, stroke severity while on a NOAC was equal to stroke severity while on a well-controlled dose of a VKA (NIHSS score 4), but stroke severity while on a NOAC was less severe when the international normalized ratio (INR) was not within the therapeutic range (NIHSS score 6) [28]. Opposing data was reported by Nakase et al., who found a higher NIHSS score in AIS while on a NOAC compared to VKA [29]. In patients qualifying for intravenous thrombolysis (IVT) or endovascular therapy (EVT), NIHSS score values were overall high, but roughly equal with NOAC and VKA pretreatment respectively [30–33].

The ischemic lesion size on magnetic resonance imaging (MRI) was reported to be significantly smaller while on a NOAC versus a VKA [23–25,29]. Also, in comparison to AIS while on acetylsalicylic acid, the size of the infarction on apixaban was smaller while the overall infarct rate was equal [34]. The distal middle cerebral artery territory was reported to be the most location of embolic strokes [18].

#### Dosing regimen

After propensity-score matching for baseline demographic parameters and co-morbidities, it was found that there was a significantly lower adherence rate than expected among the twice-daily NOAC users. The suboptimal adherence to any dosing regimen was reported to be associated with a 50% increased hazard ratio for AIS [35].

NOAC treatment was frequently found to be interrupted before an AIE [21,36]. Furthermore, subtherapeutic dosing was reported in a relevant proportion of patients as a possible explanation for NOAC failure [15,16,24,37,38]. For example, Sakamoto et al. found an inappropriately lowered dose in about 25% of patients in their cohort [15]. Nevertheless, AIE do occur despite NOAC drug levels being well within the therapeutic range [39].

#### Etiology

Overall, there are conflicting results regarding stroke subtypes in patients with AIE while on NOAC therapy. Some authors reported that cardioembolic sources, such as intracardiac thrombus or severe congestive heart failure, might present as a frequent reason for AIE under NOAC therapy [13,19–21]. On the other hand, other authors reported a predominantly non-cardioembolic etiology for AIS while on a NOAC, such as microvascular disease [15,40]. Other etiologies for AIS while on NOAC therapy include paraneoplastic coagulation disorders, arterio-arterial embolism [41] and polypharmacy [16].

#### Coagulation testing

Routine coagulation tests in patients undergoing NOAC therapy show strong variability and therefore cannot sufficiently predict current anticoagulant effect [42,43]. High plasma levels may remain elevated for more than 12 hours after last intake [43]. Despite this fact, specific coagulation tests are only performed in less than half of acute stroke patients in the emergency setting, even in experienced stroke centers [27,39].

### Reperfusion therapies in the acute setting in patients with AIS under NOAC

#### IVT

In the Get With The Guidelines register, rates of IVT use were much lower in NOAC patients compared to subtherapeutic VKA patients in the (3.3% vs. 11.7%) [28]. Also, Purrucker et al. found NOAC treatment to be a significant barrier to IVT initiation and a cause of an overall lower rate of IVT usage (9/159 patients; 5.7%) in patients on NOAC therapy [44]. Suspected or proven NOAC treatment was reported to be the main reason for not administering IVT in more than half of all patients on their register. Also, an overall time delay to treatment of 35 minutes was reported in NOAC patients compared to VKA patients [30]. In the pooled cohort, the overall rate of IVT in most tertiary stroke centers was low (5.1%) and time from symptom onset to IVT was almost two and a half hours. Seiffge et al. could show, that more than half of patients on rivaroxaban could be candidates for IVT because of low plasma levels on admission [45].

#### Deciding for or against IVT

In the absence of specific coagulation testing, the time of the last NOAC dose prior to the AIS is of major importance in the decision to administer or withhold IVT, considering also the presence or absence of drug interactions as well as renal and hepatic function. Seiffge et al. reported that about half of their patients with an AIS had their last dose less than 12 hours before hospital admission, whereas the other half had their last dose between 12 and 24 hours before hospital admission [30]. Factor IIa activity assays are the standard of care in dabigatran users with an AIS in deciding for or against IVT. However, if not available, a thrombin time (TT) based protocol (< 38 sec) might be a reasonable alternative [46]. Seiffge et al. showed that a specific factor Xa-activity assay is useful in patients treated with rivaroxaban with an AIE, and that the use of IVT in patients with low levels of factor Xa-activity might be safe. Despite using specific coagulation testing, a a fast door-to-needle time of 37 minutes was feasible [47].

#### Hemorrhagic complications after IVT

Two studies found no increase in acute-phase hemorrhagic transformation compared to VKA [15,48]. In the largest register dataset available, patients with NOAC pretreatment had the lowest unadjusted rates of life-threatening or serious systemic hemorrhage (0.4%) and of any other IVT complication (6.8%) compared to patients with antiplatelet, VKA or no medical pretreatment. However, data on coagulation parameters, the timing of the last NOAC intake, and whether nonspecific reversal strategies may have been applied were not available [49].

Olivera et al. reported ICH in two of seven patients taking a NOAC and receiving IVT. However, the definition of ICH was not clear [14]. Chen et al. reported severe bleeding events in 2/19 patients receiving IVT in patients taking rivaroxaban with both events occurring in patients with last drug intake <48h before IVT treatment [50]. In contrast, Seiffge et al. found a similar rate of symptomatic ICH using the National Institute of Neurological Disorders and Stroke (NINDS) or European Cooperative Acute Stroke Study II (ECASS-II) definition (sICH) in NOAC patients receiving IVT [30,51,52] and no sICH when the selection of therapy was guided by plasma levels in patients taking rivaroxaban [47]. Suzuki et al. reported no sICH at 24 hours in 71 patients receiving IVT with the study being limited by recall bias and reduced IVT dose [53].

#### Idarucizumab

In the biggest case series including 55 patients, IVT after dabigatran reversal was feasible with a symptom onset to needle time of 175 minutes [33]. Furthermore, its use was effective with a mean clinical improvement seen in 82% of the patients (6.3 points difference in NIHSS) and a follow-up mRS <2 in 56% of patients. The rate of sICH (3/55, 5.5%) was within the expected range. Other complications included one fatal thrombotic adverse occurring five days after the Idarucizumab/IVT infusion [54]. However, most authors reported favourable outcome of this approach [55–57].

#### Endovascular therapy (EVT)

A target large vessel occlusion was more often observed when NOAC dose was incorrectly low or compliance inadequate [58]. Seiffge et al. reported EVT without IVT in patients with high rivaroxaban plasma levels to be safe and feasible [30,47]. Several authors reported EVT to be safe and feasible with a similar rate of sICH as compared to patients without anticoagulation [59–63].

#### Outcome

Similar outcomes between AIE on NOAC or on VKA treatment were found [24]. This was also true in patients receiving IVT [30] and EVT [14,59]. Overall, in-hospital mortality was highest in patients not receiving antithrombotic treatment prior to the AIS (9.3%), followed by subtherapeutic warfarin (8.8%), antiplatelet treatment only (8.1%), therapeutic warfarin (6.4%), and NOAC (6.3%) [28].

#### Management

There are no randomized trials on the therapeutic management after AIE on NOAC. Following the AIE, switching temporarily to low molecular weight heparin was preferred by some authors [14], whereas most authors preferred switching to another NOAC with a different mechanism of action [40] for long-term prophylaxis. However, sometimes restarting the same NOAC was favored—possibly due to the good outcome of AIE on dabigatran and the possibility of antagonizing its effect by idarucizumab [33,54,64]. Also, a change of VKA was reported [14].

### Synthesis of results

The pooled data is shown in Table 1. Additionally, in S3 Table we provide the pooled data considering only studies of at least moderate quality, although there were no relevant differences compared to the data of all included studies.

**Table 1:** Pooled characteristics of patients suffering acute cerebral ischemic events while taking non-vitamin K antagonist oral anticoagulants (N = 12247).

Characteristic	item positive	item available	%	Weighted mean
Female sex	5594	10898	51.3	
Age (years)		10989		78.6
BMI (kg/m^2^)		750		26.3
**Medical history**				
Atrial fibrillation	11036	11301	97.7	
Hypertension	8614	10450	82.4	
Dyslipidemia/hyperlipidemia	4984	9721	51.3	
Previous ischemic stroke or TIA	4465	10191	43.8	
Coronary heart disease or myocardial infarction	3438	10040	34.2	
Diabetes mellitus	3456	10436	33.1	
Heart failure	2013	9616	20.9	
Smoker	745	9298	8.0	
Peripheral vascular disease	575	8900	6.5	
Carotid stenosis	415	8859	4.7	
Prosthetic heart valve	142	8768	1.6	
**NOAC**				
Rivaroxaban (total)	1626	3092	52.6	
Dabigatran (total)	1162	3092	37.6	
Apixaban (total)	299	3092	9.7	
Edoxaban (total)	5	3092	0.2	
Twice daily	635	1150	55.2	
Once daily	500	1150	43.5	
**Medication**				
Antihypertensive	7170	9190	78.0	
Cholesterol lowering drug	5866	9587	61.2	
Diabetes medication	2054	8859	23.2	
Concomitant antiplatelet	333	1668	20.0	
**Laboratory**				
Serum creatinine (mg/dl)		833		0.95
Renal clearance (ml/min)		897		63.5
apTT (sec)		650		34.5
INR		9756		1.2
Blood glucose (mg/dl)		742		124.2
D-Dimer (ng/ml)		128		964.1
BNP (pg/ml)		128		198.4
**Clinical features**				
Stroke severity (NIHSS score)		10291		4.6
**Treatment**				
Any IVT	598	9196	6.5	
Onset of symptoms to IVT (min)		402		141.9
Time since last drug intake to IVT (h)		276		10.2

BMI: body mass index, apTT: activated Partial Thromboplastin Time, INR: international normalized ratio, BNP: brain natriuretic peptide, NIHSS: National Institute of Health Stroke Severity, IVT: intravenous thrombolysis.

### Additional analysis

No additional analysis was performed.

## Discussion

### Epidemiology

It was expected that about 1% of AIS patients will be on NOAC treatment in the future [65]. With an estimated AIE rate of 1–2% per year in patients taking a NOAC and the increasing number of indications for NOAC use, this rate is likely to increase significantly [66] reaching 6% in 2017 in our tertiary stroke center.

### Risk factors and reasons for AIE under NOAC

Information on stroke subtype distribution according to validated classifications such as the Trial of Org 10172 in Acute Stroke Treatment (TOAST) or ASCOD in AIE under NOAC is currently not available [67,68].

Apart from the classical etiologies, there are several potential reasons for an AIE while taking a NOAC, including non-adherence to medication, under-dosage and potential treatment failure of NOAC with thrombus formation despite treatment.

The finding that classical vascular risk factors are associated with AIE under NOAC suggests that large artery disease or small vessel disease may represent a cause of AIE in a relevant percentage of patients [69]. However, incorporation of biomarkers associated with cardioembolic etiology (Cardiac troponin I, N-terminal pro-B-type natriuretic peptide, and D-dimer) enhanced risk assessment for subsequent ischemic events [70]. Surprisingly, the finding that previous AIS predicts treatment failure is in contrast to a subgroup analysis of the ROCKET-AF trial [71]. Future research should address the question whether impaired renal function is an independent predictor of AIE in patients taking NOAC or only if inadequate dosing is prescribed. Importantly, it was shown, that excellent renal function was a risk factor of AIE, at least in patients receiving Edoxaban [72].

Recurrent AIE is reported to occur more frequently in the first three months after NOAC therapy initiation, which behooves caregivers and patients to be especially attentive to the signs of an AIE. The elevated risk in the first weeks after the event at the same time of NOAC initiation might explain this observation. However, it might also be possible that the increased incidence in the first three months may be because NOACs may induce the mobilization of preexisting thrombi. Comparison of NOAC initiation for primary and secondary prophylaxis could clarify this phenomenon. Insufficient drug levels due to varying drug interactions have been analyzed [73], but their relevance in the real-world setting remains unclear.

Furthermore, early termination in up to one of three NOAC patients within the first year of treatment has been reported [74,75]. In this setting, mild cognitive dysfunction was not a risk factor for non-adherence [76].

### Severity and infarction size of AIE

The severity of an AIS while on a NOAC seems to be at least equal compared to VKA pretreatment. This finding is confirmed by another study that showed an equally reduced frequency of severe strokes in patients on therapeutic doses of a VKA as well as patients on NOAC therapy [77]. There is insufficient data on AIS size, although preliminary data, which showed smaller infarct volumes on MRI, was reassuring [25].

### Dosing regimen

Once daily dosing seems to be associated with a slightly better adherence [78]; although its effect in clinical practice is unclear because worse pharmacokinetics could easily balance this benefit [79]. Because body weight and renal function might be altered in the acute event setting, NOAC doses should be reviewed after about three weeks [38]. Treatment discontinuation in about a quarter of patients at one year demands care models increasing the delivery of continuous therapy [80].

### Laboratory parameters

Abnormal routine coagulation tests may provide a hint that a patient presenting with an AIE might take a NOAC, but they do not reflect the actual coagulation status. Specific tests for the activity of a particular NOAC should be used routinely, because they aid in the decision to administer or withhold IVT. Prospective studies with consistent data are warranted to define reliable recommended cut-off values for each NOAC.

### Reperfusion therapies in the acute setting in patients with AIS under NOAC

#### IVT

There are no randomized trials concerning IVT in the setting of an AIS under a NOAC. Seiffge et al. estimated that IVT could be used in more than half of patients with prior rivaroxaban treatment with about 28% of patients denied by current guidelines [30,45,81]. Recent analyses concluded that IVT is probably effective and safe in select AIS patients undergoing NOAC treatment [81–84]. However, optimal patient selection is dependent on coagulation parameters that have not yet been sufficiently evaluated, and the variable selection criteria used in available studies do not allow for a consensus [39,42,85,86]. Prospective, randomized studies are crucial for the creation of valid guidelines for this situation comparable to the INR cutoff in VKA patients.

#### EVT

EVT also seems to be safe and effective in large vessel occlusion (LVO) in patients undergoing NOAC therapy, but further trials are needed to confirm general safety [60,87]. Either on its own or in combination with, recanalization strategies are available for about one out of three patients with an AIS undergoing therapy with a NOAC [47].

#### NOAC reversal agents

Initial reports on successful and safe IVT after idarucizumab administration are promising, but limited experience does not allow for a final statement on the safety of IVT after antagonization [33,84]. Additionally, it is not clear whether idarucizumab/IVT should be administered in every dabigatran patient with an AIS or only if direct EVT is not possible. No data is presently available for the recently approved reversal agent for factor Xa inhibitors, andexanet alfa.

#### ICH

The incidence of ICH after an AIS with or without IVT/EVT seems to be low, but systematic prospective trials are lacking. Furthermore, heterogeneous definitions of a symptomatic ICH impair the comparability of study results.

#### Management

Since guidelines on the management of an AIE under NOAC therapy are lacking, and the underlying etiology is heterogeneous, it seems to be most important to search for potentially treatable sources of stroke, such as symptomatic atherosclerotic stenosis, paraneoplastic syndrome, vasculitis or endocarditis. Moreover, a measurement of NOAC activity on admission may help to identify instances of compliance-related AIE and to guide individualized dosing of NOACs in the future.

When none of the listed etiologies are found, it is controversial, but many clinicians switch to another NOAC substance [88].

### Summary of evidence

AIE in patients using a NOAC are becoming a more frequent event. Contributing risk factors as well as the etiology remain poorly understood. Stroke severity seems to be favourable or at least equal to an AIS that occurs while on a VKA. Standardized management protocols for diagnostic work-up and management are necessary to provide optimal and rapid stroke care. IVT and EVT seem to be safe and effective, but patient selection for these therapies is challenging.

### Limitations

Our analysis has several limitations that limit the generalization of findings. Those include the overall low quality of published data, statistical pooling procedures, incomplete retrieval of identified research, reporting bias, and the small number of reports of patients suffering from an AIE while using Apixaban and Edoxaban. Furthermore, we only report characteristics of patients suffering AIE while taking NOAC, but we had no information on patients on NOAC not suffering AIE which limits the explanatory power of the findings.

### Conclusions

There is an unmet need for prospective trials on the diagnostic workup and treatment of an AIE under NOAC therapy. High volume registers of patients suffering from an AIE while undergoing NOAC therapy are already being created and will hopefully soon contribute to the management of AIE under NOAC therapy. (RASUNOA prime, ClinicalTrials: NCT02533960; NOACISP; ClinicalTrials: NCT02353585, ARAMIS Register, ClinicalTrials: NCT02478177).

#### Studies included

[12–16,18–24,26–29,31,34,37,40–42,44,46,47,49,54–56,58,59,64,77,78,83,89–101].

## Supporting information

S1 Table Extracted data of included studies. Extracted items are shown.
(XLSX)

S2 Table Bias assessment tool. For each study, the risk of bias was thoroughly assessed at the study level using a modified National Heart, Lung, and Blood Institute (NHLBI) bias tool recommended for non-randomized observational studies.
(XLSX)

S3 Table Pooled characteristics of patients suffering acute cerebral ischemic events according to study quality. Data is presented of all included patiens suffering acute cerebral ischemic events while taking non-vitamin K oral anticoagulants (N = 12247) and only considering studies of at least moderate quality (n = 10840).
(DOCX)

S4 Table Prisma checklist. Checklist of systematic reviews according to the PRISMA guidelines.
(DOC)

## References

[1] Endres M, Diener H, Behnke M, Röther J, Daniels W, Dichgans M, et al. S3-Leitlinie Sekundärprophylaxe ischämischer Schlaganfall und transitorische ischämische Attacke [Internet]. [cited 19 Oct 2017]. Available: https://www.dgn.org/images/red_leitlinien/LL_2014/PDFs_Download/030-133_lang_S3_Sekundärprophylaxe_ischämischer_Schlaganfall_2015-02.pdf

[2] You JJ, Singer DE, Howard PA, Lane DA, Eckman MH, Fang MC, et al Antithrombotic Therapy for Atrial Fibrillation. Chest. 2012;141: e531S–e575S. 10.1378/chest.11-2304 22315271 PMC3278056

[3] Verma A, Cairns JA, Mitchell LB, Macle L, Stiell IG, Gladstone D, et al 2014 focused update of the Canadian Cardiovascular Society Guidelines for the management of atrial fibrillation. Can J Cardiol. 2014;30: 1114–30. 10.1016/j.cjca.2014.08.001 25262857

[4] Kirchhof P, Benussi S, Kotecha D, Ahlsson A, Atar D, Casadei B, et al 2016 ESC Guidelines for the management of atrial fibrillation developed in collaboration with EACTS. Eur Heart J. 2016;37 10.1093/eurheartj/ehw210 27567408

[5] Almutairi AR, Zhou L, Gellad WF, Lee JK, Slack MK, Martin JR, et al Effectiveness and Safety of Non-Vitamin K Antagonist Oral Anticoagulants for Atrial Fibrillation and Venous Thromboembolism: A Systematic Review and Meta-Analyses. Clin Ther. 2017;39: 1456–1478.e36. 10.1016/j.clinthera.2017.05.358 28668628

[6] Granger CB, Alexander JH, McMurray JJV V, Lopes RD, Hylek EM, Hanna M, et al Apixaban versus Warfarin in Patients with Atrial Fibrillation. N Engl J Med. 2011;365: 981–992. 10.1056/NEJMoa1107039 21870978

[7] Giugliano RP, Ruff CT, Braunwald E, Murphy SA, Wiviott SD, Halperin JL, et al Edoxaban versus warfarin in patients with atrial fibrillation. N Engl J Med. 2013;369: 2093–2104. 10.1056/NEJMoa1310907 24251359

[8] Patel MR, Mahaffey KW, Garg J, Pan G, Singer DE, Hacke W, et al Rivaroxaban versus Warfarin in Nonvalvular Atrial Fibrillation. N Engl J Med. Massachusetts Medical Society; 2011;365: 883–891. 10.1056/NEJMoa1009638 21830957

[9] Connolly SJ, Ezekowitz MD, Yusuf S, Eikelboom J, Oldgren J, Parekh A, et al Dabigatran versus Warfarin in Patients with Atrial Fibrillation. N Engl J Med. Massachusetts Medical Society; 2009;361: 1139–1151. 10.1056/NEJMoa0905561 19717844

[10] Hozo SP, Djulbegovic B, Hozo I. Estimating the mean and variance from the median, range, and the size of a sample. BMC Med Res Methodol. 2005;5: 13 10.1186/1471-2288-5-13 15840177 PMC1097734

[11] NHLBI. Quality Assessment Tool for Observational Cohort and Cross-Sectional Studies [Internet]. [cited 19 Sep 2018]. Available: https://www.nhlbi.nih.gov/health-topics/study-quality-assessment-tools

[12] Defelipe-Mimbrera A, Cánovas AA, Guillán M, Matute C, Sainz De La Maza S, Cruz A, et al Dabigatran in Secondary Stroke Prevention: Clinical Experience with 106 Patients. BioMed Research International. 2014 p. 6 10.1155/2014/567026 25133166 PMC4123474

[13] Seki K., Koga M, Yamagami H, Arihiro S, Nagatsuka K, Minematsu K, et al Clinical severity and timing of onset for acute ischemic stroke and TIA during oral anticoagulation using warfarin or non-vitamin K antagonist oral anticoagulants. Stroke. 2016.

[14] Olivera P, Gavin O, Rivero E, Constans M, Marzo C, Perea G, et al Acute Ischemic Events in Patient Receiving Direct Oral Anticoagulants for Atrial Fibrilation: Incidence, Outcome and Clinical Profile. Blood. 2016;128: 1442 LP-1442.

[15] Sakamoto Y, Okubo S, Nito C, Suda S, Matsumoto N, Abe A, et al The relationship between stroke severity and prior direct oral anticoagulant therapy in patients with acute ischaemic stroke and non-valvular atrial fibrillation. Eur J Neurol. 2017;24: 1399–1406. 10.1111/ene.13405 28799181

[16] Antoniou T, Macdonald EM, Yao Z, Hollands S, Gomes T, Tadrous M, et al Association between statin use and ischemic stroke or major hemorrhage in patients taking dabigatran for atrial fibrillation. CMAJ. 2017;189: E4–E10. 10.1503/cmaj.160303 28246253 PMC5224945

[17] Piccini JP, Stevens SR, Chang Y, Singer DE, Lokhnygina Y, Go AS, et al Renal Dysfunction as a Predictor of Stroke and Systemic Embolism in Patients With Nonvalvular Atrial Fibrillation—Clinical Perspective. Circulation. 2013;127: 224–232. 10.1161/CIRCULATIONAHA.112.107128 23212720

[18] Kamal H, Smith K, Mowla A, Shirani P, Sawyer R Jr., Fanous A, et al Ischemic Stroke in Patients with Elevated Body Mass Index while on Novel Anticoagulants: A case series report. Neurology. 2015;84: 1526–632X.

[19] Arihiro S, Todo K, Koga M, Yamagami H, Terasaki T, Kimura K, et al Three-month outcomes in Japanese stroke/TIA patients with non-valvular atrial fibrillation after initiating oral anticoagulants: The samurai-NVAF study. Int J Stroke. 2016;11: 565–574. 10.1177/1747493016632239 26927811

[20] Endo H, Kamiyama K, Mikamoto M, Takahira K, Nomura R, Honjo K, et al Acute cerebrovascular syndrome and intracranial hemorrhage in patients taking new oral anticoagulants: A single center experience. Cerebrovascular Diseases. Karger; p. Supplement 1.

[21] Hayashi T, Kato Y, Fukuoka T, Deguchi I, Maruyama H, Horiuchi Y, et al Clinical Features of Ischemic Stroke during Treatment with Dabigatran: An Association between Decreased Severity and a Favorable Prognosis. Intern Med. 2015;54: 2433–2437. 10.2169/internalmedicine.54.4948 26424298

[22] Sano H, Deguchi I, Fukuoka T, Nagamine Y, Mizuno S, Horiuchi Y.-S., et al Ischemic stroke in patients having NOACs show better prognosis than in ones having warfarin. Clin Neurol. 2016;56: S342.

[23] Shibata Y, Nakamura A, Yasaka M, Kuwashiro T, Gotoh S, Takaguchi G, et al Neurological severity and infarct size in patients with acute ischemic stroke during DOAC treatment for nonvalvular atrial fibrillation. Stroke. 2017;48: Supplement 1.

[24] Kanai Y, Oguro H, Tahara N, Matsuda H, Takayoshi H, Mitaki S, et al Analysis of Recurrent Stroke Volume and Prognosis between Warfarin and Four Non–Vitamin K Antagonist Oral Anticoagulants’ Administration for Secondary Prevention of Stroke. J Stroke Cerebrovasc Dis. 2018;27: 338–345. 10.1016/j.jstrokecerebrovasdis.2017.09.007 29033229

[25] Oguro H, Mizuhara R, Abe S, Takayoshi H, Mitaki S, Onoda K, et al Analysis of Recurrent Stroke Volume between VKA (Vitamin K Antagonist) and Three NOACs (Non-Vitamin K Antagonist Oral Anticoagulants) under Oral Anticoagulant Therapy. Int J Pharm Sci Res. 2016;3 10.15344/2394-1502/2016/116 International

[26] Piccardi B, Fratangelo R, Lamassa M, Nencini P, Pescini F. Ischemic stroke in patients treated with anticoagulants: Retrospective analysis from hospital-based register. Eur Stroke J. B. Piccardi, Careggi University Hospital, Stroke Unit, Florence, Italy; 2018;3: 469 10.1177/2396987318770127 LK - http://sfx.metabib.ch/sfx_locater?sid=EMBASE&issn=23969881&id=doi:10.1177%2F2396987318770127&atitle=Ischemic+stroke+in+patients+treated+with+anticoagulants%3A+Retrospective+analysis+from+hospital-based+register&stitle=Eur.+Stroke+J.&title=European+Stroke+Journal&volume=3&issue=1&spage=469&epage=&aulast=Piccardi&aufirst=B.&auinit=B.&aufull=Piccardi+B.&coden=&isbn=&pages=469-&date=2018&auinit1=B&auinitm=

[27] Valente M, Leung S, Wu P, Dewey H, Choi P. Acute ischaemic stroke and TIA whilst on anticoagulants—clinical characteristics and functional outcomes in the era of direct oral anticoagulants. Int J Stroke. M. Valente, Department of Neuroscience, Eastern Health, Box Hill, Australia; 2018;13: 12 10.1177/1747493018778666 LK - http://sfx.metabib.ch/sfx_locater?sid=EMBASE&issn=17474949&id=doi:10.1177%2F1747493018778666&atitle=Acute+ischaemic+stroke+and+TIA+whilst+on+anti-coagulants-clinical+characteristics+and+functional+outcomes+in+the+era+of+direct+oral+anti-coagulants&stitle=Int.+J.+Stroke&title=International+Journal+of+Stroke&volume=13&issue=1&spage=12&epage=&aulast=Valente&aufirst=Michael&auinit=M.&aufull=Valente+M.&coden=&isbn=&pages=12-&date=2018&auinit1=M&auinitm=

[28] Xian Y, O’Brien EC, Liang L, Xu H, Schwamm LH, Fonarow GC, et al Association of Preceding Antithrombotic Treatment With Acute Ischemic Stroke Severity and In-Hospital Outcomes Among Patients With Atrial Fibrillation. JAMA. 2017;317: 1057 10.1001/jama.2017.1371 28291892

[29] Nakase T, Moroi J, Ishikawa T. Difference of Clinical Condition of Ischemic Stroke between Warfarin and Direct Oral Anticoagulants (P6.285). Neurology. 2017;88: Supplement P6.285.

[30] Seiffge DJ, Hooff R-J, Nolte CH, Bejot Y, Turc G, Ikenberg B, et al Recanalization therapies in acute ischemic stroke patients: impact of prior treatment with novel oral anticoagulants on bleeding complications and outcome. Circulation. 2015;132: 1261–1269. 10.1161/CIRCULATIONAHA.115.015484 26232277

[31] Kimura S, Ogata T, Fukae J, Okawa M, Higashi T, Iwaasa M, et al Revascularization for acute ischemic stroke is safe for rivaroxaban users. J Stroke Cerebrovasc Dis. Elsevier Ltd; 2014;23: e427–e431. 10.1016/j.jstrokecerebrovasdis.2014.05.015 25149204

[32] Pikija S, Sztriha LK, Sebastian Mutzenbach J, Golaszewski SM, Sellner J. Idarucizumab in Dabigatran-Treated Patients with Acute Ischemic Stroke Receiving Alteplase: A Systematic Review of the Available Evidence. CNS Drugs. 2017;31 10.1007/s40263-017-0460-x 28808918 PMC5573762

[33] Giannandrea D, Caponi C, Mengoni A, Romoli M, Marando C, Gallina A, Marsili E, Sacchini E, Mastrocola S, Padiglioni C, Mazzoli T, Cenciarelli SRS. Intravenous thrombolysis in stroke after dabigatran reversal with idarucizumab: case series and systematic review. J Neurol Neurosurg Psychiatry. 2018;7: 1–5. 10.1136/jnnp-2018-318658 30032118

[34] O’Donnell MJ, Eikelboom JW, Yusuf S, Diener HC, Hart RG, Smith EE, et al Effect of apixaban on brain infarction and microbleeds: AVERROES-MRI assessment study. Am Heart J. Elsevier B.V.; 2016;178: 145–150. 10.1016/j.ahj.2016.03.019 27502862

[35] Alberts MJ, Peacock WF, Fields LE, Bunz TJ, Nguyen E, Milentijevic D, et al Association between once- and twice-daily direct oral anticoagulant adherence in nonvalvular atrial fibrillation patients and rates of ischemic stroke. Int J Cardiol. 2016;215: 11–13. 10.1016/j.ijcard.2016.03.212 27104919

[36] Cavallari I, Ruff CT, Nordio F, Deenadayalu N, Shi M, Lanz H, et al Clinical events after interruption of anticoagulation in patients with atrial fibrillation: An analysis from the ENGAGE AF-TIMI 48 trial. Int J Cardiol. Elsevier B.V.; 2018;257: 102–107. 10.1016/j.ijcard.2018.01.065 29395361

[37] Kato Y, Hayashi T, Tanahashi N, Takao M. The Dose of Direct Oral Anticoagulants and Stroke Severity in Patients with Acute Ischemic Stroke and Nonvalvular Atrial Fibrillation. J Stroke Cerebrovasc Dis. Elsevier Inc.; 2018;27: 1490–1496. 10.1016/j.jstrokecerebrovasdis.2017.12.038 29398536

[38] Shinoda N, Mori M, Tamura S, Korosue K, Kose S, Kohmura E. Risk of Recurrent Ischemic Stroke with Unintended Low-Dose Oral Anticoagulant Therapy and Optimal Timing of Review. J Stroke Cerebrovasc Dis. Elsevier Inc.; 2018;27: 1546–1551. 10.1016/j.jstrokecerebrovasdis.2018.01.002 29395644

[39] Purrucker JC, Haas K, Rizos T, Khan S, Poli S, Kraft P, et al Coagulation Testing in Acute Ischemic Stroke Patients Taking Non–Vitamin K Antagonist Oral Anticoagulants. Stroke. 2017;48: 152–158. 10.1161/STROKEAHA.116.014963 27899756

[40] Cappellari M, Bovi P. Continuation of direct oral anticoagulants in the acute phase of ischemic stroke. A case series. J Thromb Thrombolysis. Springer US; 2017;43: 248–251. 10.1007/s11239-016-1430-8 27699550

[41] Kim BJ, Kang HG, Lee DH, Kang D-W, Kim JS, Kwon SU. Ischemic Stroke on Optimal Anticoagulation with Novel-Oral Anticoagulants Compared with Warfarin. Int J Stroke. 2015;10: E68–E68. 10.1111/ijs.12587 26202718

[42] Kepplinger J, Prakapenia A, Barlinn K, Siegert G, Gehrisch S, Zerna C, et al Standardized use of novel oral anticoagulants plasma level thresholds in a new thrombolysis decision making protocol. J Thromb Thrombolysis. 2015;41 10.1007/s11239-015-1229-z 26001908

[43] Seiffge D, Kägi G, Zedde M, Bejot Y, Turc G, Michel P, et al Rivaroxaban plasma levels in patients with acute ischemic stroke and intracerebral hemorrhage. Eur Stroke J. 2017;2: 99–100. 10.1177/2396987317705242 29394504

[44] Purrucker JC, Haas K, Rizos T, Khan S, Poli S, Kraft P, et al Coagulation testing in acute ischemic stroke patients taking non-Vitamin K antagonist oral anticoagulants. Stroke. 2017;48: 152–158. 10.1161/STROKEAHA.116.014963 27899756

[45] Seiffge DJ, Kägi G, Michel P, Fischer U, Béjot Y, Wegener S, et al Rivaroxaban plasma levels in acute ischemic stroke and intracerebral hemorrhage. Ann Neurol. 2018;83: 451–459. 10.1002/ana.25165 29394504

[46] Kate M, Szkotak A, Witt A, Shuaib A, Butcher K. Proposed approach to thrombolysis in dabigatran-treated patients presenting with ischemic stroke. J Stroke Cerebrovasc Dis. Elsevier Ltd; 2014;23: 1351–1355. 10.1016/j.jstrokecerebrovasdis.2013.11.013 24406026

[47] Seiffge DJ, Traenka C, Polymeris AA, Thilemann S, Wagner B, Hert L, et al Intravenous Thrombolysis in Patients with Stroke Taking Rivaroxaban Using Drug Specific Plasma Levels: Experience with a Standard Operation Procedure in Clinical Practice. J Stroke. 2017;19: 1–10. 10.5853/jos.2016.00024 28877563 PMC5647628

[48] Purrucker JC, Haas K, Wolf M, Rizos T, Khan S, Kraft P, et al Haemorrhagic Transformation after Ischaemic Stroke in Patients Taking Non-vitamin K Antagonist Oral Anticoagulants. J Stroke. 2017;19: 67–76. 10.5853/jos.2016.00542 28178406 PMC5307942

[49] Xian Y, Federspiel JJ, Hernandez AF, Laskowitz DT, Schwamm LH, Bhatt DL, et al Use of Intravenous Recombinant Tissue Plasminogen Activator in Patients With Acute Ischemic Stroke Who Take Non–Vitamin K Antagonist Oral Anticoagulants Before StrokeClinical Perspective. Circulation. 2017;135: 1024–1035. 10.1161/CIRCULATIONAHA.116.023940 28119380

[50] Chen ST, Hellkamp AS, Becker RC, Berkowitz SD, Breithardt G, Fox KAA, et al Outcome of Patients Receiving Thrombolytic Therapy While on Rivaroxaban for Nonvalvular Atrial Fibrillation (from Rivaroxaban Once Daily Oral Direct Factor Xa Inhibition Compared With Vitamin K Antagonism for Prevention of Stroke and Embolism Trial in Atr. Am J Cardiol. Elsevier Inc.; 2017;120: 1837–1840. 10.1016/j.amjcard.2017.07.095 28886856

[51] Hacke W, Kaste M, Fieschi C, von Kummer R, Davalos A, Meier D, et al Randomised double-blind placebo-controlled trial of thrombolytic therapy with intravenous alteplase in acute ischaemic stroke (ECASS II). Second European-Australasian Acute Stroke Study Investigators. Lancet. Elsevier; 1998;352: 1245–51. 10.1016/S0140-6736(98)08020-9 9788453

[52] National Institute of Neurological Disorders and Stroke rt-PA Stroke Study Group. Tissue Plasminogen Activator for Acute Ischemic Stroke. N Engl J Med. 1995;333: 1581–1588. 10.1056/NEJM199512143332401 7477192

[53] Suzuki K, Aoki J, Sakamoto Y, Abe A, Suda S, Okubo S, et al Low risk of ICH after reperfusion therapy in acute stroke patients treated with direct oral anti-coagulant. J Neurol Sci. Elsevier B.V.; 2017;379: 207–211. 10.1016/j.jns.2017.06.004 28716241

[54] Kermer P, Eschenfelder CC, Diener H-C, Grond M, Abdalla Y, Althaus K, et al Antagonizing dabigatran by idarucizumab in cases of ischemic stroke or intracranial hemorrhage in Germany–A national case collection. Int J Stroke. 2017;12: 1747 10.1177/1747493017701944 28494694

[55] Pikija S, Sztriha LK, Sebastian Mutzenbach J, Golaszewski SM, Sellner J. Idarucizumab in Dabigatran-Treated Patients with Acute Ischemic Stroke Receiving Alteplase: A Systematic Review of the Available Evidence. CNS Drugs. 2017;31: 747–757. 10.1007/s40263-017-0460-x 28808918 PMC5573762

[56] Šaňák D, Jakubíček S, Černík D, Herzig R, Kunáš Z, Mikulík R, et al Intravenous Thrombolysis in Patients with Acute Ischemic Stroke after a Reversal of Dabigatran Anticoagulation with Idarucizumab: A Real-World Clinical Experience. J Stroke Cerebrovasc Dis. 2018;27: 2479–2483. 10.1016/j.jstrokecerebrovasdis.2018.05.004 29807757

[57] Frol S, Pretnar Oblak J. Safe and very effective intravenous thrombolysis after idarucizumab application in stroke patients receiving dabigatran therapy: A case series. Int J Stroke. S. Frol, University Clinical Centre Ljubljana, Department of vascular neurology, Ljubljana, Slovenia; 2018;13: 218–219. 10.1177/1747493018789543 LK - http://sfx.metabib.ch/sfx_locater?sid=EMBASE&issn=17474949&id=doi:10.1177%2F1747493018789543&atitle=Safe+and+very+effective+intravenous+thrombolysis+after+idarucizumab+application+in+stroke+patients+receiving+dabigatran+therapy%3A+A+case+series&stitle=Int.+J.+Stroke&title=International+Journal+of+Stroke&volume=13&issue=2&spage=218&epage=219&aulast=Frol&aufirst=S.&auinit=S.&aufull=Frol+S.&coden=&isbn=&pages=218-219&date=2018&auinit1=S&auinitm=

[58] Woo H, Han M. Patterns of stroke recurrence in ischemic stroke patients taking non-vitamin K antagonist oral anticoagulation. Cerebrovasc Dis. H.G. Woo, Soonchunhyang University Cheonan Hospital, South Korea; 2018;46: 23 10.1159/000493155 LK - http://sfx.metabib.ch/sfx_locater?sid=EMBASE&issn=14219786&id=doi:10.1159%2F000493155&atitle=Patterns+of+stroke+recurrence+in+ischemic+stroke+patients+taking+non-vitamin+K+antagonist+oral+anticoagulation&stitle=Cerebrovasc.+Dis.&title=Cerebrovascular+Diseases&volume=46&issue=&spage=23&epage=&aulast=Woo&aufirst=Ho+Geol&auinit=H.G.&aufull=Woo+H.G.&coden=&isbn=&pages=23-&date=2018&auinit1=H&auinitm=G

[59] Rebello LC, Haussen DC, Belagaje S, Anderson A, Frankel M, Nogueira RG. Endovascular treatment for acute ischemic stroke in the setting of anticoagulation. Stroke. 2015;46: 3536–3539. 10.1161/STROKEAHA.115.011285 26470775

[60] Zapata-Wainberg G, Ximénez-Carrillo Á, Trillo S, Fuentes B, Cruz-Culebras A, Aguirre C, et al Mechanical thrombectomy in orally anticoagulated patients with acute ischemic stroke. J Neurointerv Surg. 2018;10: 834–838. 10.1136/neurintsurg-2017-013504 29275325

[61] Krajíčková D, Vyšata O, Čabelková P, Halúsková S, Vališ M, Vítková E, et al Safety and efficacy of mechanical thrombectomy with stent-retrievers in anticoagulated patients with anterior circulation stroke. Clin Radiol. 2018;74: 165.e11-165.e16. 10.1016/j.crad.2018.10.009 30420266

[62] D. Č, D. Š, P. D, M. K, F. C, J. Z, et al Mechanical Thrombectomy in Patients with Acute Ischemic Stroke on Anticoagulation Therapy. Cardiovasc Intervent Radiol. 2018;41: 706–711. 10.1007/s00270-018-1902-7 29450625

[63] Kurowski D, Jonczak K, Shah Q, Yaghi S, Marshall RS, Ahmad H, et al Safety of Endovascular Intervention for Stroke on Therapeutic Anticoagulation: Multicenter Cohort Study and Meta-Analysis. J Stroke Cerebrovasc Dis. Elsevier Inc.; 2017;26: 1104–1109. 10.1016/j.jstrokecerebrovasdis.2016.12.027 28110890

[64] Nedkova Hristova V, De Felipe Mimbrera A, Escribano-Paredes B, Martinez Poles J, Garcia Madrona S, Perez Torre R, et al Clinical characteristics of ischemic stroke in patients treated previously with direct oral anticoagulants. Eur J Neurol. V. Nedkova Hristova, Neurology, Hospital Universitario Ramón y Cajal, Madrid, Spain; 2018;25: 389.

[65] Pfeilschifter W, Farahmand D, Niemann D, Ikenberg B, Hohmann C, Abruscato M, et al Estimating the Quantitative Demand of NOAC Antidote Doses on Stroke Units. Cerebrovasc Dis. 2016;42: 415–420. 10.1159/000447952 27438461

[66] Pollack C V. Evidence Supporting Idarucizumab for the Reversal of Dabigatran. Am J Med. Elsevier; 2016;129: S73–S79. 10.1016/j.amjmed.2016.06.008 27568285

[67] Adams H., Bendixen B., Kappelle L., Biller J, Love B., Gordon D., et al Classification of Subtype of Acute Ischemic Stroke. Stroke. 1993;23: 35–41. 10.1161/01.STR.24.1.35 7678184

[68] Amarenco P, Bogousslavsky J, Caplan LR, Donnan GA, Wolf ME, Hennerici MG. The ASCOD phenotyping of ischemic stroke (Updated ASCO Phenotyping). Cerebrovasc Dis. 2013;36: 1–5. 10.1159/000352050 23899749

[69] Elvira-Ruiz G, Caro-Martinez C, Andreu-Cayuelas J, Flores-Blanco P, H A-I, Gomez-Molina M, et al Comparison of thromboembolic and bleeding risk scores in direct oral anticoagulant naive patients with non-valvular atrial fibrillation. Eur Heart J. G. Elvira-Ruiz, University Hospital Virgen De La Arrixaca, Murcia, Spain; 2017;38: 967. 10.1093/eurheartj/ehx502.P4575 LK - http://sfx.metabib.ch/sfx_locater?sid=EMBASE&issn=15229645&id=doi:10.1093%2Feurheartj%2Fehx502.P4575&atitle=Comparison+of+thromboembolic+and+bleeding+risk+scores+in+direct+oral+anticoagulant+naive+patients+with+non-valvular+atrial+fibrillation&stitle=Eur.+Heart+J.&title=European+Heart+Journal&volume=38&issue=&spage=967&epage=&aulast=Elvira-Ruiz&aufirst=G.&auinit=G.&aufull=Elvira-Ruiz+G.&coden=&isbn=&pages=967-&date=2017&auinit1=G&auinitm=

[70] Ruff CT, Giugliano RP, Braunwald E, Murphy SA, Brown K, Jarolim P, et al Cardiovascular biomarker score and clinical outcomes in patients with atrial fibrillation: A subanalysis of the ENGAGE AF-TIMI 48 randomized clinical trial. JAMA Cardiol. 2016;1: 999–1006. 10.1001/jamacardio.2016.3311 27706467

[71] Hankey GJ, Patel MR, Stevens SR, Becker RC, Breithardt G, Carolei A, et al Rivaroxaban compared with warfarin in patients with atrial fibrillation and previous stroke or transient ischaemic attack: A subgroup analysis of ROCKET AF. Lancet Neurol. 2012;11: 315–322. 10.1016/S1474-4422(12)70042-X 22402056

[72] Yu HT, Yang P-S, Joung B. Impact of Renal Function on Outcomes With Edoxaban in Real-World Patients With Atrial Fibrillation. Stroke. 2018;49: 2421–2429. 10.1161/STROKEAHA.118.021387 30355093

[73] Xiong Q, Lau YC, Lip GYH. Pharmacodynamic profile and drug interactions with non-vitamin K antagonist oral anticoagulants: implications for patients with atrial fibrillation. Expert Opin Drug Metab Toxicol. 2015;11: 937–948. 10.1517/17425255.2015.1027683 25797167

[74] Jackevicius CA, Tsadok MA, Essebag V, Atzema C, Eisenberg MJ, Tu J V, et al Early non-persistence with dabigatran and rivaroxaban in patients with atrial fibrillation. Heart. 2017;1: 1–8. 10.1136/heartjnl-2016-310672 28286333

[75] Forslund T, Wettermark B, Hjemdahl P. Comparison of treatment persistence with different oral anticoagulants in patients with atrial fibrillation. Eur J Clin Pharmacol. 2016;72: 329–338. 10.1007/s00228-015-1983-z 26613954

[76] Horstmann S, Rizos T, Saribas M, Efthymiou E, Rauch G, Veltkamp R. Cognitive Impairment is Not a Predictor of Failure to Adhere to Anticoagulation of Stroke Patients with Atrial Fibrillation. Cerebrovasc Dis. 2015;39: 325–331. 10.1159/000381728 25966900

[77] Hellwig S, Grittner U, Audebert H, Endres M, Haeusler KG. Non-vitamin K-dependent oral anticoagulants have a positive impact on ischaemic stroke severity in patients with atrial fibrillation. Europace. 2017; 1–6. 10.1093/europace/euw133 28460024 PMC5889015

[78] Alberts MJ, Peacock WF, Fields LE, Bunz TJ, Nguyen E, Milentijevic D, et al Association between once- and twice-daily direct oral anticoagulant adherence in nonvalvular atrial fibrillation patients and rates of ischemic stroke. Int J Cardiol. Elsevier Ireland Ltd; 2016;215: 11–13. 10.1016/j.ijcard.2016.03.212 27104919

[79] Heidbuchel H, Vrijens B, Gross R, Andrade A, Lalama C, Eshleman S. Non-vitamin K antagonist oral anticoagulants (NOAC): considerations on once- vs. twice-daily regimens and their potential impact on medication adherence. Europace. Oxford University Press; 2015;17: 1317–1318. 10.1093/europace/euv124 26045506

[80] Kirchhof P, Radaideh G, Kim YH, Lanas F, Haas S, Amarenco P, et al Global Prospective Safety Analysis of Rivaroxaban. J Am Coll Cardiol. 2018;72: 141–153. 10.1016/j.jacc.2018.04.058 29976287

[81] Seiffge DJ, Polymeris AA, Fladt J, Lyrer PA, Engelter ST, De Marchis GM. Management of patients with stroke treated with direct oral anticoagulants. J Neurol. Springer Berlin Heidelberg; 2018;0: 0. 10.1007/s00415-018-9061-y 30293111

[82] Tsivgoulis G, Safouris A. Intravenous Thrombolysis in Acute Ischemic Stroke Patients Pretreated with Non-Vitamin K Antagonist Oral Anticoagulants. Stroke. 2017;48: 2031–2033. 10.1161/STROKEAHA.117.017206 28536167

[83] Suzuki K, Aoki J, Sakamoto Y, Abe A, Suda S, Okubo S, et al Low risk of ICH after reperfusion therapy in acute stroke patients treated with direct oral anti-coagulant. J Neurol Sci. Elsevier B.V; 2017;379: 207–211. 10.1016/j.jns.2017.06.004 28716241

[84] Jin C, Huang RJ, Peterson ED, Laskowitz DT, Hernandez AF, Federspiel JJ, et al Intravenous tPA (Tissue-Type Plasminogen Activator) in Patients With Acute Ischemic Stroke Taking Non–Vitamin K Antagonist Oral Anticoagulants Preceding Stroke. Stroke. 2018;49: 2237–2240. 10.1161/STROKEAHA.118.022128 30354981 PMC6706353

[85] Steiner T, Böhm M, Dichgans M, Diener HC, Ell C, Endres M, et al Recommendations for the emergency management of complications associated with the new direct oral anticoagulants (DOACs), apixaban, dabigatran and rivaroxaban. Clin Res Cardiol. 2013;102: 399–412. 10.1007/s00392-013-0560-7 23669868

[86] Touzé E, Gruel Y, Gouin-Thibault I, De Maistre E, Susen S, Sie P, et al Intravenous thrombolysis for acute ischemic stroke in patients on direct oral anticoagulants. Eur J Neurol. 2018;25: 747–e52. 10.1111/ene.13582 29360254

[87] Liu M, Zheng Y, Li G. Safety of Recanalization Therapy in Patients with Acute Ischemic Stroke Under Anticoagulation: A Systematic Review and Meta-Analysis. J Stroke Cerebrovasc Dis. Elsevier Inc.; 2018;27: 2296–2305. 10.1016/j.jstrokecerebrovasdis.2018.04.012 30017747

[88] Rybinnik I, Mullen MT, Messe S, Kasner SE, Cucchiara B. Treatment of acute stroke in patients on Dabigatran: A survey of US stroke specialists. J Stroke Cerebrovasc Dis. Elsevier Ltd; 2013;22: 1312–1316. 10.1016/j.jstrokecerebrovasdis.2012.12.005 23313461

[89] Vornicu O, Larock A-S, Dincq A-S, Douxfils J, Dogné J-M, Mullier F, et al Idarucizumab for the treatment of hemorrhage and dabigatran reversal in patients requiring urgent surgery or procedures. Expert Opin Biol Ther. 2017;17: 1275–1296. 10.1080/14712598.2017.1349749 28728489

[90] Volbers B, Köhrmann M, Kallmünzer B, Kurka N, Breuer L, Ringwald J, et al Dabigatran Plasma Levels in Acute Cerebrovascular Events. J Stroke Cerebrovasc Dis. 2016;25: 877–882. 10.1016/j.jstrokecerebrovasdis.2015.12.024 26809705

[91] Tse DM, Young L, Ranta A, Barber P. Intravenous alteplase and endovascular clot retrieval following reversal of dabigatran with idarucizumab. JNNP. 2017; 1–2. 10.1136/jnnp-2017-316449 28986468

[92] Tomita H, Hagii J, Metoki N, Saito S, Shiroto H, Hitomi H, et al Severity and Functional Outcome of Patients with Cardioembolic Stroke Occurring during Non-vitamin K Antagonist Oral Anticoagulant Treatment. J Stroke Cerebrovasc Dis. W.B. Saunders; 2015;24: 1430–1437. 10.1016/j.jstrokecerebrovasdis.2015.03.004 25843224

[93] Tabata E, Yasaka M, Wakugawa Y, Okada Y. Recombinant Tissue type Plasminogen Activator (rt-PA) Therapy in an Acute Stroke Patient Taking Dabigatran Etexilate: A Case Report and Literature Review. Intern Med. 2014;53: 2013–2015. 10.2169/intern 25030563

[94] Stöllberger C, Finsterer J. Presentation, therapy and outcome of patients with ischemic stroke under new oral anticoagulants. Neurol Neurochir Pol. 2014;48: 136–140. 10.1016/j.pjnns.2014.03.001 24821640

[95] Shahjouei S, Tsivgoulis G, Shahripour RB, Morgan Jones G, Alexandrov A V, Zand R. Safety of Intravenous Thrombolysis among Stroke Patients Taking New Oral Anticoagulants—Case Series and Systematic Review of Reported Cases. J Stroke Cerebrovasc Dis. 2015;24: 2685–2693. 10.1016/j.jstrokecerebrovasdis.2015.07.021 26542821

[96] Seiffge DJ, Traenka C, Polymeris A, Hert L, Fisch U, Peters N, et al Feasibility of rapid measurement of Rivaroxaban plasma levels in patients with acute stroke. J Thromb Thrombolysis. 2017;43: 112–116. 10.1007/s11239-016-1431-7 27696335

[97] Bluecher A, Dos Santos SM, Ferreirós N, Labocha S, Meyer Dos Santos IMR, Picard-Willems B, et al Microfluidic coagulation assay for monitoring anticoagulant therapy in acute stroke patients. Thromb Haemost. 2017;117: 519–528. 10.1160/TH16-08-0619 28124061

[98] Hoyer C, Filipov A, Neumaier-Probst E, Szabo K, Ebert A, Alonso A. Impact of pre-admission treatment with non-vitamin K oral anticoagulants on stroke severity in patients with acute ischemic stroke. J Thromb Thrombolysis. Springer US; 2018;45: 529–535. 10.1007/s11239-018-1634-1 29476304

[99] Purrucker JC, Wolf M, Haas K, Siedler T, Rizos T, Khan S, et al Microbleeds in ischemic vs hemorrhagic strokes on novel oral anticoagulants. Acta Neurol Scand. 2018;138: 163–169. 10.1111/ane.12934 29663313

[100] Shin H, Cho MC, Kim RB, Kim CH, Choi NC, Kim SK, et al Laboratory measurement of apixaban using anti-factor Xa assays in acute ischemic stroke patients with non-valvular atrial fibrillation. J Thromb Thrombolysis. Springer US; 2018;45: 250–256. 10.1007/s11239-017-1590-1 29198080

[101] Altay S. New oral anticoagulants-TURKey (NOAC-TURK): Multicenter cross-sectional study. Anatol J Cardiol. 2017; 353–361. 10.14744/AnatolJCardiol.2016.7472 28100898 PMC5469081

