# Supplementary material for: Clinical presentation, diagnostic findings and management of cerebral ischemic events in patients on treatment with non-vitamin K antagonist oral anticoagulants – A systematic review
Source: PLoS One. 2019 Mar 29;14(3):e0213379. doi: 10.1371/journal.pone.0213379 (PMC6440627; doi:10.1371/journal.pone.0213379)
Supplement: S3 Table — Data is presented of all included patiens suffering acute cerebral ischemic events while taking non-vitamin K oral anticoagulants (N = 12247) and only considering studies of at least moderate quality (n = 10840). (DOCX) [file pone.0213379.s003.docx]

|  | **Only moderate quality studies** | | | |  | **All studies included** | | | |
| --- | --- | --- | --- | --- | --- | --- | --- | --- | --- |
| **Characteristic** | **item positive** | **item available** | **%** | **Weighted mean** |  | **item positive** | **item available** | **%** | **Weighted mean** |
| Female sex | 5179 | 9813 | 52.8 |  |  | 5594 | 10898 | 51.3 |  |
| Age (years) |  | 10840 |  | 78.9 |  |  | 10989 |  | 78.6 |
| BMI (kg/m^2^) |  | 669 |  | 26.7 |  |  | 750 |  | 26.3 |
| **Medical history** |  |  |  |  |  |  |  |  |  |
| Atrial fibrillation | 9625 | 9848 | 97.7 |  |  | 11036 | 11301 | 97.7 |  |
| Hypertension | 8298 | 10013 | 82.9 |  |  | 8614 | 10450 | 82.4 |  |
| Dyslipidemia/hyperlipidemia | 4862 | 9357 | 52.0 |  |  | 4984 | 9721 | 51.3 |  |
| Previous ischemic stroke or TIA | 4239 | 9710 | 43.7 |  |  | 4465 | 10191 | 43.8 |  |
| Coronary heart disease or myocardial infarction | 3373 | 9696 | 34.8 |  |  | 3438 | 10040 | 34.2 |  |
| Diabetes mellitus | 3335 | 9999 | 33.4 |  |  | 3456 | 10436 | 33.1 |  |
| Heart failure | 1976 | 9471 | 20.9 |  |  | 2013 | 9616 | 20.9 |  |
| Smoker | 689 | 8982 | 7.7 |  |  | 745 | 9298 | 8.0 |  |
| Peripheral vascular disease | 571 | 8859 | 6.4 |  |  | 575 | 8900 | 6.5 |  |
| Carotid stenosis | 415 | 8859 | 4.7 |  |  | 415 | 8859 | 4.7 |  |
| Prosthetic heart valve | 142 | 8902 | 1.6 |  |  | 142 | 8768 | 1.6 |  |
|  |  |  |  |  |  |  |  |  |  |
| **NOAC** |  |  |  |  |  |  |  |  |  |
| Rivaroxaban (total) | 647 | 1576 | 41.1 |  |  | 1626 | 3092 | 52.6 |  |
| Dabigatran (total) | 781 | 1576 | 49.6 |  |  | 1162 | 3092 | 37.6 |  |
| Apixaban (total) | 147 | 1576 | 9.3 |  |  | 299 | 3092 | 9.7 |  |
| Edoxaban (total) | 1 | 1576 | 0.0 |  |  | 5 | 3092 | 0.2 |  |
| Twice daily | 1305 | 2167 | 60.2 |  |  | 635 | 1150 | 55.2 |  |
| Once daily | 862 | 2167 | 39.8 |  |  | 500 | 1150 | 43.5 |  |
| **Medication** |  |  |  |  |  |  |  |  |  |
| Antihypertensive | 7136 | 9190 | 77.6 |  |  | 7170 | 9190 | 78.0 |  |
| Cholesterol lowering drug | 5900 | 9587 | 61.5 |  |  | 5866 | 9587 | 61.2 |  |
| Diabetes medication | 2054 | 8859 | 23.2 |  |  | 2054 | 8859 | 23.2 |  |
| Concomitant antiplatelet | 261 | 1093 | 23.9 |  |  | 333 | 1668 | 20.0 |  |
| **Laboratory** |  |  |  |  |  |  |  |  |  |
| Serum creatinine (mg/dl) |  | 712 |  | 0.96 |  | 833 |  | 0.95 |  |
| Renal clearance (ml/min) |  | 391 |  | 65.1 |  | 897 |  | 63.5 |  |
| apTT (sec) |  | 134 |  | 33.0 |  | 650 |  | 34.5 |  |
| INR |  | 9260 |  | 1.20 |  | 9756 |  | 1.2 |  |
| Blood glucose (mg/dl) |  | 643 |  | 122.7 |  | 742 |  | 124.2 |  |
| D-Dimer (ng/ml) |  |  |  |  |  |  | 128 |  | 964.1 |
| BNP (pg/ml) |  |  |  |  |  |  | 128 |  | 198.4 |
| **Clinical features** |  |  |  |  |  |  |  |  |  |
| Stroke severity (NIHSS score) |  | 9343 |  | 4.5 |  |  | 10291 |  | 4.6 |
| **Treatment** |  |  |  |  |  |  |  |  |  |
| Any IVT |  |  |  |  |  | 598 | 9196 | 6.5 |  |
| Onset of symptoms to IVT (min) |  |  |  |  |  |  | 402 |  | 141.9 |
| Time since last drug intake to IVT (h) |  |  |  |  |  |  | 276 |  | 10.2 |

Table 1 – Pooled characteristics of patients suffering acute cerebral ischemic events under treatment with non-vitamin K oral anticoagulants.
